# Krill Oil and Its Bioactive Components as a Potential Therapy for Inflammatory Bowel Disease: Insights from In Vivo and In Vitro Studies

**DOI:** 10.3390/biom14040447

**Published:** 2024-04-06

**Authors:** Yingying Liu, Ainsley M. Robinson, Xiao Qun Su, Kulmira Nurgali

**Affiliations:** 1 Institute for Health & Sport, Victoria University, Melbourne, VIC 3021, Australia; yingying.liu1@live.vu.edu.au (Y.L.); ainsley.robinson@vu.edu.au (A.M.R.); 2 School of Rural Health, La Trobe University, Melbourne, VIC 3010, Australia; 3 Department of Medicine Western Health, The University of Melbourne, Melbourne, VIC 3010, Australia; 4 Regenerative Medicine and Stem Cells Program, Australian Institute for Musculoskeletal Science (AIMSS), Melbourne, VIC 3021, Australia

**Keywords:** inflammatory bowel disease (IBD), krill oil, long-chain n-3 polyunsaturated fatty acids (LC n-3 PUFA), eicosapentaenoic acid (EPA), docosahexaenoic acid (DHA), astaxanthin

## Abstract

Krill oil is extracted from krill, a small crustacean in the Antarctic Ocean. It has received growing attention because of krill oil’s unique properties and diverse health benefits. Recent experimental and clinical studies suggest that it has potential therapeutic benefits in preventing the development of a range of chronic conditions, including inflammatory bowel disease (IBD). Krill oil is enriched with long-chain n-3 polyunsaturated fatty acids, especially eicosapentaenoic and docosahexaenoic acids, and the potent antioxidant astaxanthin, contributing to its therapeutic properties. The possible underlying mechanisms of krill oil’s health benefits include anti-inflammatory and antioxidant actions, maintaining intestinal barrier functions, and modulating gut microbiota. This review aims to provide an overview of the beneficial effects of krill oil and its bioactive components on intestinal inflammation and to discuss the findings on the molecular mechanisms associated with the role of krill oil in IBD prevention and treatment.

## 1. Introduction

Inflammatory bowel disease (IBD), including two major chronic inflammatory disorders of the gastrointestinal (GI) tract, Crohn’s disease (CD) and ulcerative colitis (UC), is characterised by alternating phases of clinical relapse and remission [1]. In CD, inflammation is transmural, occurring with patchy “skip lesions” in any part of the GI tract from the mouth to the anus, whereas UC is distinguished by diffuse and continuous inflammation restricted to the mucosa and submucosa of the colon and rectum [2,3,4]. Whilst these two major subtypes of IBD are essentially differentiated by morphological patterns and the localisation of inflammation within the GI tract, overlapping histopathological and endoscopic features can create complexity in distinguishing CD from UC, resulting in a diagnosis of unspecified or indeterminate IBD [5,6]. The clinical presentations of all IBD subtypes are similar, including fatigue, prolonged diarrhoea with or without gross bleeding, abdominal pain, weight loss, and fever [2]. Frequent complications associated with disease progression in CD are abscess and stricture formation, intestinal obstruction, and fistulas [4]. Moreover, patients with IBD have a higher risk of developing other complications, such as asthma or bronchitis, psoriasis, pericarditis, ischemic heart disease, and colon carcinoma [7,8,9,10]. Several key factors are related to the development of IBD, including genetic components (over 240 nonoverlapping genetic risk loci), environmental elements (e.g., smoking, diet, drugs, geography, social stress, and psychological elements), microbial dysbiosis, and altered immune responses [11,12]. Thus, the cause of IBD is considered to be multifactorial—an inappropriate immune response to pathogens in the gut in a genetically predisposed host [13]. However, the exact aetiology of IBD remains unclear. 

Current IBD therapies involve conventional pharmaceutical treatments, including corticosteroids, aminosalicylates, antibiotics, and immunosuppressive therapies, which aim to alleviate symptoms and prolong remission [14]. However, these therapies have been associated with loss of response over time and severe side effects, including nausea, vomiting, heartburn, diarrhoea, headache, neutropenia, immunosuppression, and liver toxicity [15,16,17]. The application of biological therapies using monoclonal antibodies directed against specific targets implicated in the pathogenesis of IBD has increased in past years, including anti-tumour necrosis factor alpha (TNF-α), anti-integrin, and anti-interleukin (IL)-12/23 agents [14]. Other biological therapies are currently under investigation in clinical trials, such as anti-beta-7 integrin, and anti-IL-13 [18,19]. Biological therapy has fewer side effects than conventional pharmaceutical treatments and has been demonstrated as efficacious for inducing and maintaining remission in moderate-to-severe UC and CD; however, 10–40% of IBD patients fail to respond to initial treatment and up to 50% experience a loss of response over time [1,20,21,22,23]. 

In addition, approximately 25% of patients cease anti-TNF therapy due to adverse events [24,25]. Therefore, surgery is still often necessitated; 25–33% of patients require surgery within five years after biological therapy [26]. Since surgical treatment is not curative in IBD, post-operation recurrence and repeated surgery rates remain high [26,27]. Furthermore, biological therapies are expensive, require repeated intravenous infusions or subcutaneous injections, and carry long-term safety concerns, including the potential development of congestive heart failure, skin lesions, immune reactions, increased susceptibility to infections and cancers, and decreased fertility/adverse effects on pregnancy [22,28].

Given that the efficacy of current IBD treatments is limited and the burden of the disease, both on patients and healthcare systems, is substantial [29], it is imperative to develop novel therapeutic agents that are safer, cheaper, and more effective. Natural products with anti-inflammatory and antioxidant activities with lower toxicity, side effects, and cost than conventional treatments offer a potential therapeutic approach for IBD.

Omega-3 long-chain polyunsaturated fatty acids (LC n-3 PUFAs), a family of PUFAs, are characterised by the location of the first double bond between the third and fourth carbon atoms in the hydrocarbon (acyl) chain from the methyl end [30]. Fatty fish and other marine species, including krill and other shellfish, as well as algae, are the richest dietary sources of LC n-3 PUFAs [31]. Studies have suggested that LC n-3 PUFAs, especially eicosapentaenoic acid (EPA) and docosahexaenoic acid (DHA), have potent anti-inflammatory effects and a range of health benefits in cardiovascular diseases, diabetes, cancer, depression, age-related cognitive decline, and rheumatoid arthritis [32]. LC n-3 PUFAs from fish oil have shown beneficial effects on IBD, including reducing the need for corticosteroid therapy [33] and decreasing the risk of UC [34]. In addition, these FAs improved coagulation function and reduced major postoperative complications in patients with UC [35]. Fish oil enriched with LC n-3 PUFAs can lower the risk of IBD by 16% [36]. Moreover, it has also been proven to reduce inflammation in patients with IBD, accompanied by a significant reduction in high-sensitivity CRP, erythrocyte sedimentation rate, procalcitonin, and IL-6 [37]. The possible mechanisms by which LC n-3 PUFAs exert their anti-inflammatory effects include the inhabitation of arachidonic acid derivatives, the alteration of inflammatory gene expression via effects on transcription factors, and the synthesis of specialised pro-resolving mediators, such as resolvins, protectins, and maresins [38].

Krill oil (KO) is a valuable dietary supplement due to its high concentrations of LC n-3 PUFAs, EPA (13.8–20.3%), and DHA (5.6–17.4%) [39]. In the past decade, KO has received growing attention because of its unique properties and potent health benefits, including cardiovascular disease prevention, positive effects on premenstrual syndrome, and anti-obesity, anti-diabetic, anti-cancer, and neuroprotective activities [40]. KO and its bioactive components exert their beneficial effects on intestinal inflammation via anti-inflammatory and antioxidant actions, the improvement of intestinal barrier function, and the modulation of gut microbiota [41,42,43]. 

This review discusses the properties, molecular mechanisms of action, and health benefits of KO and its bioactive components in relation to IBD based on the currently available studies. The PubMed, PubMed Central, Medline, Springer Link, and Wiley Online Library databases were searched using the keywords of krill oil, inflammatory bowel disease or IBD, colitis, ulcerative colitis or UC, Crohn’s disease or CD, inflammation, n-3 PUFA, EPA, DHA, astaxanthin, and fish oil. The title and abstract were used for the initial screening. Full-text screening was then applied for peer-reviewed original research and relevant key citations. Exclusion criteria included preprints, conference proceedings, articles with only abstracts available, and articles that were not written in English.

## 2. Properties of Krill Oil 

Krill oil is extracted from krill, a small crustacean that is particularly abundant in the Northern (Arctic) and Southern (Antarctic) polar seas [44]. The largest krill species, Antarctic *Euphausia superba*, is found in the Antarctic Ocean and is currently the primary source of KO [45]. Antarctic KO has been reported to have a range of beneficial properties, including anti-inflammatory effects, modifying lipid metabolism, reducing hyperlipidaemia, attenuating oxidative stress, improving memory and neurocognitive functions, protecting the cardiovascular system, and preventing cancer development [40,41,43,46,47,48,49]. KO is unique compared to fish oil because 30–65% of its LC n-3 PUFAs are bound to phospholipids rather than triglycerides, suggesting a higher bioavailability of these fatty acids in KO [45,50,51,52]. In addition, KO contains the antioxidant astaxanthin, vitamins A and E (tocopherols), minerals, and a novel flavonoid [45]. Thus, KO may be a better source of LC n-3 PUFAs than fish oil and provide more health benefits.

## 3. The Intestinal Barrier

The intestinal mucosal barrier functions both as a semi-permeable structure that permits the absorption of water, electrolytes, and essential dietary nutrients from the gut lumen into the circulation, as well as a mediator of crosstalk between commensal gut microbes and the immune system, constituting a first line of defence against potentially harmful microorganisms, foreign antigens, and toxins [53]. To aid its functions, the intestinal mucosa is composed of several elements, including an outer mucus layer, which harbours luminal commensal gut microbiota, antimicrobial peptides (AMPs), and immunoglobulin-A (IgA); a central epithelial cell layer; and an inner lamina propria where innate and adaptive immune cells reside [53,54]. The main constituent of the mucosal barrier is the intestinal epithelium, a single layer of specialised cell subtypes interconnected by dynamic junctional complexes, such as tight junctions (TJs), which provide mechanical cohesion and the regulation of paracellular permeability [55]. 

Pattern recognition receptors (PRRs) expressed by epithelial cells, such as Toll-like receptors (TLRs) and intracellular nucleotide-binding oligomerisation domain (NOD)-like receptors (NLRs), are essential for maintaining intestinal barrier integrity by recognising microbe-specific pathogen- or damage-associated molecular patterns [56]. The detection of conserved bacteria or their components, such as membrane lipids, lipoproteins, proteins, lipopolysaccharide (LPS), and peptidoglycan (PGN), results in the ligation of TLRs and/or NLRs, initiating the activation of signalling molecules, such as nuclear factor-κB (NF-κB), and impacts the expression of proinflammatory cytokines, chemokines, and AMPs [57]. These downstream effects are significantly implicated in the regulation of pathogen eradication, commensal homeostasis, and linkage to adaptive immunity. 

### 3.1. The Intestinal Barrier and IBD

Defects in intestinal barrier function are characteristic of IBD, including a compromised mucus layer, increased epithelial barrier permeability, alterations in PRRs, adenosine monophosphate production, and autophagy processes [58]. These mucosal barrier perturbations result in inadequate protection against microbial adherence and invasion, enabling increased amounts of infiltrating bacteria to directly contact the epithelium. Serum endotoxin levels are known to correlate with both intestinal mucosal permeability and disease activity in IBD; the impairment of intestinal barrier integrity leads to increased permeability for LPSs and other bacterial toxins [59]. Consequently, compensatory immune reactions towards pathogens are excessively triggered, such as enhanced dendritic cell, macrophage, and natural killer (NK) cell activation and T-helper (Th) cell cytokine production, a process that can stimulate diverse inflammatory responses and result in and exacerbate chronic inflammation [53]. In an innate immune response, activated dendritic cells and macrophages produce pro-inflammatory cytokines, IL-1β, IL-6, IL-18, and tumour necrosis factor (TNF), which further aggravate the local inflammation initiated by immature dendritic cell IL-23 production. Furthermore, activated NK cells bind to infected epithelial cells, release toxic particles, and induce apoptosis [60]. An adaptive immune response is also generated through the dendritic cell activation of T cells to differentiate into Th1, Th2, and Th17 cells. Th1 cells are stimulated to produce interferon (IFN)-γ by IL-12 and IL-18, which play a role in the inflammatory cascade in CD, while Th2 cells release IL-4, IL-5, and IL-13, which are associated with UC [60,61].

### 3.2. Protective Roles of Krill Oil in Intestinal Barrier Impairment Associated with Intestinal Inflammation

Several studies have reported positive effects of KO treatment in maintaining intestinal barrier integrity and reducing the morphological damage induced by intestinal inflammation in animal models of colitis [62,63,64,65]. Both KO supplementation for 28 days and a 9-day KO liposome treatment ameliorated dextran sodium sulfate (DSS)-induced morphological damage to the colon, including necrosis of the intestinal wall, distortion of crypt structure, crypt loss, submucosal oedema, and the infiltration of inflammatory cells [64,65]. Additionally, goblet cell loss was attenuated by KO supplementation for 28 days in *Trichuris suis* (*T. suis*)-infected pigs [63]. Studies on DSS-induced colitis also report that KO supplementation increased the mRNA expression level of *Muc6* (Mucin 6), TJ-related genes, claudin-1, occludin, and zonula occludens (ZO)-1, and reduced serum levels of LPS and diamine oxidase (DAO), indicating a decrease in intestinal permeability [64,65,66]. In an in vitro study, KO liposomes almost fully restored the decreased transepithelial electrical resistance in LPS-treated RAW264.7 cells [65]. Additionally, in another in vitro study, scratch wound healing analyses to evaluate epithelial restitution capability showed that KO treatment for 18 h restored E-cadherin expression and decreased stress fibre formation in cells exposed to a pro-inflammatory cytokine mix [62]. This study also used bacterial adhesion, invasion, and survival assays to determine that KO reduced bacterial adhesiveness and the invasiveness of adherent-invasive *Escherichia coli* (AIEC). Collectively, these studies demonstrate the potential of KO supplementation to inhibit the inflammatory response and maintain the integrity of the intestinal barrier by promoting epithelial functional and morphological restitution, reducing intestinal barrier permeability, and exerting a direct effect on the invasion of harmful symbiotic bacteria.

## 4. Immune Response in Intestinal Inflammation

Available evidence suggests that the dysfunctions in innate and adaptive immune pathways contribute to aberrant intestinal inflammatory responses in patients with IBD [67]. Genome-wide association studies (GWAS) have identified susceptibility loci *IL23R*, *IL12B* (*p40*), *JAK2,* and *STAT3* in both UC and CD patients [68,69]. Pro-inflammatory cytokines IL 12 and IL 23 belong to the IL 12 family of cytokines, which provide an important regulatory link between innate and adaptive immunity. *IL12B*, which encodes the p40 subunit of IL-12 and IL-23 that binds to the IL 12Rβ1 chain, induces the activation of the JAK–STAT pathway [70]. IL-12 activates the differentiation of T cells into interferon (IFN)-γ producing Th1 cells and natural killer (NK) cells to secrete IFN-γ and TNF-α [71], while IL-23 induces the proliferation and survival of Th17 cells, which can secrete high amounts of IL-17 and IL-17F, along with TNF-α, IL-6, IL-21, and IL-22 [72]. As mentioned previously, TLRs on the cell surface and NLRs in the cytoplasm are important for recognising microbial antigens [67]. The binding of LPS to TLR4 induces TLR4 signalling to activate NF-κB and mitogen-activated protein kinases (MAPKs), which in turn causes an increased proliferation and differentiation of macrophages and the expression of pro-inflammatory cytokines, such as TNF-α, IL-6, and IL-12 [73,74].

The emergence of biological therapies for IBD is a result of an increased understanding of the role of inflammatory pathways in IBD pathogenesis. Biological therapies use monoclonal antibodies targeting TNF-α, integrins, and cytokine molecules [28]. Anti-TNF-α biologics neutralise TNF-α-mediated pro-inflammatory signalling by binding to homotrimeric TNF-α and blocking the interaction between the cytokine and TNF-α receptor types 1 and 2 [73]. Integrins are transmembrane receptors located on inflammatory cells that assist in cell adhesion, signalling, and migration. The mechanism of action for biological anti-integrin agents is to block integrins and thereby interfere with the migration of leukocytes to the site of inflammation [75]. The most recent class of biological therapy for IBD is the anti-IL-12/23 antibody, which binds to the shared p40 subunit of IL-12 and IL-23 heterodimers, inhibiting the interaction of these cytokines with their cognate receptors [76]. While demonstrated as highly efficacious for moderate-to-severe UC and CD, biological therapies are expensive, and need to be administered intravenously or subcutaneously; in addition, proteolytic GI enzymes can destroy them, and most patients either do not respond to initial treatment or lose responsiveness over time [1,77].

### Anti-Inflammatory Effects of Krill Oil in Intestinal Inflammation 

Several studies have shown that KO can attenuate inflammation in DSS-induced colitis by exerting anti-inflammatory effects to regulate the release of pro-inflammatory cytokines, including TNF-α, IL-1β, and IL-6, and elevate the expression of anti-inflammatory cytokine IL-10 [78,79], as shown in Table 1. Moreover, KO in combination with anti-inflammatory compounds—either celecoxib (cyclooxygenase-2 inhibitor) or TPCA1 (IκB kinase 2 inhibitor)—resulted in a further reduction in the expression of pro-inflammatory genes, such as *IL-6*, *NOD2*, and chemokine ligand 2 (*CCL2*) over KO alone [63]. This suggests that the combination of KO with other anti-inflammatory agents may potentially enhance the anti-inflammatory effects of KO. According to RNA sequencing-based transcriptome analyses, KO can inhibit a range of LPS-activated pathways, including cytokine–cytokine receptor interactions and NF-κB, chemokine, NLR, TLR, peroxisome proliferator-activated receptor (PPAR), and TNF signalling pathways [63].

It has been reported that KO emulsion can inhibit LPS-induced pro-inflammatory activation in macrophages in a dose-dependent manner by inhibiting the specific ultra-pure LPS activation of TLR4. As a result, the expression of TNF-α is also reduced [80]. Another study demonstrated that KO reduces inflammation in DSS-induced colitis by inhibiting the activation of the NF-κB signalling pathway [64]. Furthermore, FlexPro-MD, a mixture of KO, astaxanthin, and hyaluronic acid, specifically inhibits the NF-κB signalling pathway induced by LPS via reducing phosphorylation levels of NF-κB p65 and inhibitor of κB-α (IκB-α) [81].

KO may facilitate the resolution phase of inflammation by facilitating the M1 to M2 polarisation of human macrophages. It has been reported that KO supplementation decreases multiple M1 macrophage marker genes (*CCL2, IL12B, CXCL9, CXCL10, CXCL11,* and *CD80*) and restores the expression of LPS-inhibited M2 macrophage markers (EGR2 and CD36) in vitro [63]. The same study also showed that KO supplementation for 17 days ameliorates both initial and pro-resolving phases of inflammation in *Citrobacter rodentium* (*C. rodentium*)-induced colitis in mice, enhancing macrophage phagocytosis and macrophage-mediated intracellular bacterial killing. KO supplementation decreased mucosa-attached *C. rodentium* bacterial load, resulting in a significant decrease in the expression of pro-inflammatory cytokines, such as TNF-α, IL-1β, IL-12, IL-17A, IL-22, and CCL2, while increasing metabolites involved in pro-resolvin pathways, including aspirin-triggered resolvin E and lipoxin biosynthesis pathways [63]. Another in vitro study showed that KO downregulated the AIEC-induced mRNA expression of pro-inflammatory cytokines IL-8 and TNFα in epithelial cells [62]. Therefore, the possible mechanism associated with the anti-inflammatory effect of KO in animal models of colitis includes the regulation of pro-inflammatory cytokine production and NF-κB transcriptional activity. Taken together, these studies suggest that KO treatment may hinder the activation of the NF-κB signalling pathway via the inhibition of IκB-α and p65 phosphorylation and a subsequent reduction in the expression of downstream inflammatory factors to relieve intestinal inflammation.

Currently, the primary therapeutic strategies for IBD are mainly focused on the initial phase of inflammation with anti-inflammatories, corticosteroids, immunosuppressants, or biologicals. While this approach can be effective in managing acute GI inflammation that causes symptoms, it may not comprehensively address the chronic aspects of the condition. Experimental studies suggest KO as an alternative IBD treatment approach that focuses on the resolution phase of inflammation by enhancing the body’s natural resolution mechanisms, reducing dependency on pharmaceutical interventions and minimising side effects. However, clinical studies are necessitated to confirm the anti-inflammatory effects of KO in IBD and to further evaluate its underlying molecular mechanisms.

## 5. Oxidative Stress

Reactive oxygen species (ROS) are produced in cellular response to xenobiotics, cytokines, and bacterial invasion, as well as by-products of oxidative metabolism mainly during mitochondrial respiration [82]. Cellular redox homeostasis is maintained by a comprehensive endogenous antioxidant defence system in aerobic organisms [83]. The term oxidative stress refers to the state of imbalance between ROS production and the capacity of this defence system to generate an efficient response in favour of oxidants [84]. At low-to-moderate concentrations, ROS functions as a second messenger, signalling molecules to regulate cellular physiological and biological processes; however, the antioxidant defence system can become overwhelmed by the excessive production of ROS, resulting in redox imbalance, which subsequently disrupts cellular integrity and functions, including damage to lipids, proteins, and DNA [85]. 

### 5.1. Oxidative Stress and IBD

Reduced antioxidant levels and increased quantities of biomarkers for oxidative stress and ROS-mediated damage have been demonstrated in intestinal tissue from IBD patients and animal models of colitis [86,87,88]. A recent meta-analysis identified 438 differentially expressed oxidative stress genes between CD and healthy control tissues, highlighting that the role of oxidative stress as a key pathophysiological mechanism to CD is regulated by DNA methylation and host–microbiota interactions [89]. Excessive ROS production by intestinal epithelial cells, as well as neutrophils and macrophages, can damage cytoskeleton proteins and initiate alterations in TJs and epithelial permeability during mucosal inflammation. This allows luminal pathogen invasion and leukocyte infiltration, resulting in barrier disruption [90,91]. Subsequently, cumulative damage to the intestinal tissue results in mucosal necrosis and ulceration, characteristic of IBD [92,93]. Mucosal NADPH oxidases (NOX), such as the NOX2 complex, NOX1, and dual oxidase 2 (DUOX2), which participate in endogenous ROS generation by catalysing chemical reactions, are activated during inflammation to produce greater amounts of ROS, and thus have been reported as novel IBD risk factors [91]. Nuclear factor-erythroid 2-related factor 2 (Nrf2) is a key transcription factor that can maintain mucosa homeostasis by suppressing excessive ROS generation in IBD. Under oxidative stress conditions, Nrf2 translocates to the nucleus and binds to the antioxidant response elements (AREs), leading to the expression of antioxidant enzymes, including glutathione-S-transferase (GST), heme oxygenase-1 (HO-1), NADP(H) quinone oxidoreductase 1 (NQO1), and catalase (CAT) [94]. Nrf2-deficient mice were reported to be more susceptible to DSS-induced colitis than wild-type mice, indicated by enhanced rectal bleeding, colon shortening, crypt overgrowth, and increased immune cell infiltration [95]. More recently, in an IL-10 knockout model of chronic colitis, reduced mucosal damage and prolapse rates were observed in response to Nrf2 activation. However, in the same study, acute DSS-induced colitis was aggravated in genetically modified mice expressing constitutively active NRF2 selectively in epithelial and myeloid cells [96]. These experimental data suggest that both Nrf2 deficiency and overexpression can aggravate disease phenotype. In addition, there may be different roles of Nrf2 activation during acute and chronic inflammation, indicating the importance of the tight regulation of Nrf2 during the onset of intestinal inflammation [97].

### 5.2. Antioxidant Effects of Krill Oil in Inflammatory Conditions

Several studies have shown the antioxidant potential of KO treatment in physiological and pathological conditions, including colitis [78,98,99]. Dietary supplementation of 5% KO for four weeks significantly reduced levels of oxidative stress markers, carbonyl glutamic semialdehyde, and glycoxidation products (carboxyethyl lysine (CEL) and carboxymethyl lysine (CML)) in distal colon tissue from rats with DSS-induced colitis [78]. Further investigations are required to determine whether the antioxidant effects of KO reported in this study may be due to the astaxanthin component of KO or, given that PPAR-γ and PPAR-γ coactivator 1α (Pparg1α) expression was increased following KO supplementation, the n-3 PUFAs from KO, as PPAR-γ agonists can block oxidative damage from advanced glycation end products like CML and CEL [78,100]. A KO diet of 80 mg/kg per day for four weeks decreased superoxide anion production in brain tissue from LPS-induced Alzheimer’s disease mice. It was considered that KO decreases amyloidogenesis and memory deficiency via the prevention of brain damage caused by oxidative stress and neuroinflammation [99]. The neuroprotective effect of KO on oxidative stress has also been demonstrated in an experimental cuprizone (CPZ) model of multiple sclerosis [101]. In this study, a diet supplemented with 5% KO for 13 weeks reduced the malondialdehyde (MDA) level and increased the superoxide dismutase (SOD) activity, glutathione peroxidase (GSH-Px) activity, and glutathione (GSH) content in the corpus callosum of CPZ mice. Furthermore, KO treatment decreased the levels of Kelch-like ECH-associated protein l (KEAP1) and increased Nrf2 protein expression, suggesting that KO activated the KEAP1/Nrf2 antioxidant pathway [101]. Similarly, KO has also been reported to protect dopaminergic cells against oxidative stress by increasing SOD and GSH-Px activities and decreasing nitric oxide production [102]. In an in vitro study, ROS production by human immortalised keratinocytes was inhibited after KO treatment for 24 h [98]. While the evidence for the antioxidant effects of KO is currently limited, these studies imply the therapeutic potential of KO in oxidative-stress-associated pathological conditions.

## 6. Modulation of Gut Microbiota by Krill Oil

The composition and balance of intestinal microbiota are related to intestinal health. Bacteria from the *Firmicutes* and *Bacteroidetes* phyla represent 90% of the gut microbiota and the *Firmicutes*/*Bacteroidetes* (F/B) ratio is closely related to intestinal homeostasis [103]. Changes in this ratio can result in various pathologies, and an increasing abundance of specific *Firmicutes* or *Bacteroidetes* species can lead to obesity and bowel inflammation, respectively [104]. Several studies have shown that the abundance and composition of gut microbiota are altered in IBD patients, with a decrease in microbial biodiversity and total number of species compared to healthy individuals [6,105,106,107]. Furthermore, the diversity of the gut microbiome can be used for the differential diagnosis of CD and UC, while abundance of mucosal bacteria is associated with the severity of the disease [107].

KO may reduce the severity of colitis through the regulation of the structural composition of the gut microbiota. In mice with DSS-induced colitis, it has been demonstrated that 0.5 g/kg KO treatment can restore the F/B ratio, increasing *Firmicutes*, *Proteobacteria*, and *Epsilonbacteraeota,* and decreasing *Bacteroidetes* and *Patescibacteria*. KO reversed changes to the abundance of *Bacteroides, Alisties*, *Paraprevotella*, *Candidatus Arthromitus*, *Ruminiclostridium* 9, the *Rikenellaceae* RC9 gut group, and the [*Eubacterium*] *nodatum* group at the genus level [64]. Likewise, 1.5 g/day KO for 28 days significantly increased gut microbial diversity and restored microbial interactions in a porcine model of *T. suis* infection [63]. The abundance of genus *Pasteurella* and *Candidatus Savagella* increased in pigs fed with KO, while *Lactobacillus* and an unclassified genus in *Rickettsiales* were reduced. In the same study, 1.5 mg KO administered orally for 17 days in mice with *C. rodentium*-induced colitis reversed the genus level increases in *SMB53*, *Erwinia*, and *Bifidobacterium* induced by infection. Another study showed that KO decreases the adhesion and invasion of AIEC strain LF82 to epithelial cells [62]. Furthermore, KO significantly reduced the proliferation and survival of LF82 in macrophages after 18 h of treatment.

The intestinal metabolome of IBD patients is disordered and is characterised by an imbalance of short-chain fatty acids (SCFAs), bile acids, and tryptophan [108]. A primary source of energy for colon cells, SCFAs have a significant impact on intestinal homeostasis, energy metabolism, and immune response modulation [109]. Levels of SCFAs, butyrate, propionate, and acetate indirectly reflect the metabolic activities of intestinal microbiota, which have long been believed to preserve the intestinal epithelium integrity in IBD inflammation [110,111]. Butyrate can induce innate lymphocytes (ILCs) to produce AMPs involved in the regulation of microbiota composition [112]. In a study that analysed the composition and abundance of SCFAs following 0.5g/kg KO supplementation for four weeks in DSS-induced mice, it was demonstrated that KO regulates the production of SCFAs and the metabolic pathway, and modulates the relationship between SCFAs and the gut microbiota [64]. KO counteracted the declining trend of acetic acid, propionic acid, isobutyric acid, butyric acid, isovaleric acid, and valeric acid. Correspondingly, the relative abundance of butyric acid and butyrate-producing bacteria *Odoribacter*, *Butyricicoccus*, *Roseburia*, and *Clostridium* increased following treatment with KO [64]. KO had a significant impact on tryptophan metabolism, significantly affecting multiple metabolites, such as 5-hydroxy-L-tryptophan and 5-hydroxyindoleacetic acid [66]. Histamine drives the severity of innate inflammation in experimental colitis models [113]. In pigs infected with *T. suis*, KO supplementation reduced several key metabolites related to histidine metabolism by suppressing the expression of a gene encoding L-histidine decarboxylase (HDC) in the colonic mucosa and decreasing histamine biosynthesis of microbial origin [63]. Since certain strains of *Lactobacillus reuteri* (*L. reuteri*) contain a gene cluster encoding HDC, and the abundance of *L. reuteri* and several other *Lactobacillus* species was reduced by KO in this study, the authors postulated that KO may regulate histamine of microbial origin via suppressing *Lactobacillus* abundance. KO also significantly enriched *Anaeroplasmataceae*, *Clostridiaceae*, and *Christensenellaceae* at the family level, which were all negatively correlated with the level of valyl-histidine in the *C. rodentium*-induced colitis model [66]. These results suggest that the consumption of KO can help regulate the microbial metabolites in the intestine via regulation of the gut microbiota, thereby contributing to the attenuation of colitis. Therefore, KO supplementation may be a potential microbe-based therapeutic approach for IBD; however, further studies are necessary to support this hypothesis.

In summary, all reported animal studies investigating the effectiveness of KO on intestinal inflammation have used experimental models with induced intestinal inflammation by chemicals, parasites, or bacteria. While the outcomes from these studies are promising, it would be more relevant for future clinical translation if animal models with spontaneous chronic intestinal inflammation, such as Winnie mice, were included. This animal model carries a missense mutation in Muc2 and has previously been demonstrated to be highly representative of human IBD [114,115,116]. Data from those studies would help us to understand the potential clinical efficacy of KO in IBD and associated molecular mechanisms.

## 7. Clinical Studies Investigating the Effects of Fish Oil and the Status of Krill Oil on IBD

Clinical studies with fish oil and its LC n-3 PUFAs have shown a range of beneficial effects on IBD, although few discrepant findings have been reported [34,36,37,117,118]. A meta-analysis and systemic review of observational studies has shown an inverse association between fish oil consumption and the risk of CD. Similarly, such association was also found between the intake of LC n-3 PUFAs and the risk of UC [34]. In large-cohort population-based studies, the regular consumption of oily fish and/or habitual intake of fish oil supplements was associated with a lower risk of IBD [119]. In contrast, another systematic review and meta-analysis of randomised controlled trials reported that a high LC n-3 PUFA intake may increase the risk of developing IBD and increase faecal calprotectin levels, a specific inflammatory marker for IBD, but may reduce the risk of IBD relapse and disease worsening [120]. Inconsistencies in these clinical outcomes may be related to various factors such as sample size, gender and age of the participants, and the dosage and duration of supplementation, as well as the condition and severity of disease. The synergistic clinical effects of fish oil in combination with other agents have also been reported in previous studies. An oral supplement enriched with fish oil, fructooligosaccharides, gum arabic, vitamin E, vitamin C, and selenium was found to reduce corticosteroid requirements in patients with mild-to-moderate UC [121]. In addition, a combination of 5-ASA and LC n-3 FAs has been shown to effectively maintain remission in paediatric CD [122].

A 2023 meta-analysis of 90 RCTs (59,940 participants) evaluated the safety and tolerability of n-3 PUFA supplements and found them to be generally well tolerated with no evidence of serious adverse events [123]. Nonserious adverse events that were more common in participants taking n-3 PUFAs, compared with the control group, were diarrhoea, dysgeusia, and bleeding tendencies, as well as abnormal laboratory values (elevated alanine transaminase, platelet and blood urea nitrogen, decreased alkaline phosphatase, tissue plasminogen activator haemoglobin, haematocrit, mean arterial pressure, and C-reactive protein). Similarly, in a study involving 60 UC patients in remission, the administration of EPA (0.5 g/day) for six months did not result in serious adverse effects. Only one patient discontinued the study due to bloating, and another due to diarrhoea [124]. The risk of atrial fibrillation and GI symptoms (diarrhoea, nausea, dyspepsia, abdominal discomfort) in statin-treated participants with high cardiovascular risk were increased with high-dose EPA/DHA (4 g/day) supplements [125]. Currently, the European Food Safety Authority (EFSA) suggests the consumption of LC n-3 PUFA is not associated with adverse effects in healthy children or adults, and the long-term consumption of EPA and DHA supplements at combined doses of up to 5000 mg/day is safe [126].

As reported earlier, KO has unique biochemical properties compared with fish oil. This implies it may be more beneficial for IBD patients. However, large-scale randomised clinical trials are warranted to validate the efficacy of KO on IBD and assess its effectiveness compared with existing clinical therapies. This is important as the currently available treatments for IBD are associated with toxicity, severe side effects, and loss of response. Proven to induce remission and prevent relapse, 5-ASAs are also associated with the exacerbation of IBD symptoms and serious side effects, such as pancreatitis, pleuritis, myocarditis, and nephritis [127,128]. While corticosteroids are highly effective for inducing remission, they demonstrate toxicity effects and a loss of response over time, and contribute to major infection [14,129,130]. Therapy with immunomodulators is the mainstay treatment for moderate-to-severe CD and active UC where 5-ASA or corticosteroid treatments have failed; however, the associated toxicity is highly variable and unpredictable among patients and long-term use is correlated with an increased rate of infection and cancer risk [130,131,132]. Biological therapies are demonstrated to be highly efficacious for moderate-to-severe IBD; nonetheless, many patients either do not respond to initial treatment or lose responsiveness over time [1,20,21,22,23]. In addition, biological therapies are costly and have been associated with the reactivation of serious infections, such as tuberculosis and hepatitis B, as well as an increased risk of cancer [14,22,28]. Based on the positive outcomes of fish and fish oil consumption in IBD patients, especially with little or no side effects observed, and the available data from experimental models of intestinal inflammation with KO supplementation, it is plausible that KO may be an advantageous agent for IBD management and treatment. According to the animal study by Zhou et al. [64] and using the method of Nair and Jacob [133], the equivalent human dosages of KO for IBD treatment would be 20–40 mg/kg body weight/day. This indicates that, for a young male adult with approximately 70 kg body weight, 1.4–2.8 g of KO would be required daily [6]. These dosages are feasible for many individuals. However, validation in clinical trials is required as outcomes from animal studies are not directly translated to humans.

**Table 1:** A summary of studies demonstrating the effects of krill oil in experimental animal models of IBD and other diseases.

Animal Models	Inducer	Treatment	Concentration	Treatment Method	Duration	Effects	Reference
DSS-induced colitis in 24 male BALB/c mice	3.5% DSS in drinking water	KO	Low: 0.25 g/kg High: 0.5 g/kg	Gavage	21 days: 14-day treatment prior to and 7-day co-treatment with DSS	0.25 g/kg KO: NO change in body weight and colon length, IL-6,TNF-α, LPS, DAO ↓Histological damage, ↑ZO-1, ↑Occludin, ↓p-p65, ↓p-IκB-α 0.5 g/kg KO: ↓Body weight loss, ↓Disease activity index, ↑Colon length, ↓Histological damage, ↓IL-6, ↓TNF-α, ↑ZO-1, ↑Occludin, ↑Claudin-1, ↓LPS, ↓DAO, ↓p-p65, ↓p- IκB-α, ↑Gut microbiota uniformity and richness, ↑*Firmicutes* to *Bacteroidetes* ratios, ↑SCFAs	[64]
DSS-induced colitis in 30 male Wistar rat	5% DSS in drinking water	KO	5% KO	Mixed with diets	28 days: 21-day treatment prior to and 7-day co-treatment with DSS	↓Disease activity index, ↑Colon length, ↓Histological damage, ↓TNF-α, ↓KC/GRO, ↓IL-1β, ↑PGE_2_, ↑PGE_3_, ↑PGD_2_, ↑EPA, ↑DHA, ↓n-6 FAs, ↓GSA, ↓CEL, ↓CML, ↑Ppargc1 a, ↑Pparg, ↑Sirt 1, ↑TLR4, ↓*Nos2*, ↓IL-6, ↓KC/GRO, ↓*Ptgs*, ↓*Ptgs2*	[78]
DSS-induced colitis in 20 female C57BL/6 mice	3% DSS in drinking water	KLD (KO+VitD3+LR *Lactobacillus reuteri*)	16 mg/per mouse	Gavage	7 days: co-treatment with DSS	↓Body weight loss, ↑Colon length, ↓Histological damage, ↓TNF-α, ↓IL-1β, ↓IL-6, ↑IL-10	[134]
DSS-induced colitis in 48 male C57BL/6 mice	2% DSS in drinking water	KO liposomes	300 μM/kg	Gavage	9 days: 4-day treatment prior to and 5-day co-treatment with DSS	↓Disease activity index, ↑Colon length, ↓Histological damage, ↓TNF-α, ↓IL-6, ↓LPS	[65]
DSS-induced colitis in 50 male C57BL/6 mice	3% DSS in drinking water	Antarctic krill phospholipids (APL)	10, 25, 50 mg/kg	Gavage	7 days: co-treatment with DSS	10 mg/kg APL: No change, Body weight loss, Colon weight, Histological damage, Disease activity index, TNF-α, IL-6, ZO-1, ↑Occludin 25 and 50 mg/kg APL: ↓Body weight loss, ↑Colon weight, ↓Histological damage, ↓Disease activity index, ↓TNF-α,↓IL-6, ↑ZO-1, ↑Occludin, 50 mg/kg APL: No change in Chao index of OTU level, ↑*Firmicutes/Bacteroidetes* ratio, ↑SCFAs (Acetic acid; Propionic acid; Butyric acid)	[79]
40 parasitic whipworm *T. suis*-infected pigs	Parasitic whipworm *T. suis* eggs by feeding	KO	1.5 g daily	Mixed in a freshly made sugar-coated cookie dough ball	28 days: 7-day treatment prior to and 21-day treatment co-treatment with Parasitic whipworm *T. suis*	↓Crypt length, ↑Goblet cell numbers, ↑Histological scores, ↑Microbial alpha diversity indices, ↑*Firmicutes* to *Bacteroidetes* ratios, ↓*Rickettsiales* abundance, ↓*Lactobacillus vaginalis* species abundance, ↓L-histidine decarboxylase and gut histamine, ↓1-methylhistamine and N-acetylhistamine	[63]
30 *C. rodentium* infected male C3H/HeNCr mice	*C. rodentium* (bacteria) by oral gavage	KO	1.5 mg daily	Gavage	17 days: 5-day treatment prior to and 12-day co-treatment with *C. rodentium*	↓Body weight loss, ↓Histological damage, ↓Load of *C. rodentium*, improved the microbial dysbiosis index, ↓*Lactobacillus reuteri*, *L. vaginalis*, *Clostridium perfringens* abundance, *↓ IL-1β*, *↓TNF*, *↓IL-12B*, *↓IL-17A*, *↓IL-22*, *↓CCL2*	[63]
DSS-induced colitis in 50 C57BL/6 mice	3% DSS dissolved in physiological saline and free drinking	KO-HIPPE (high internal phase Pickering emulsion)	200 mg/kg 400 mg/kg 800 mg/kg	Gavage	9 days co-treatment with DSS	200 mg/kg KO-HIPPE: ↑Colon length, ↓Histological damage, ↓TNF-α,↓IL-6, MDA, SOD 400 mg/kg KO-HIPPE: ↑Colon length (No significant difference), ↓Histological damage, ↑Occludin, ↓TNF-α,↓IL-6, MDA, ↑GSH, SOD 800 mg/kg KO-HIPPE: ↑Colon length, ↓Histological damage, ↓TNF-α,↓IL-6, MDA,	[135]
36 *C. rodentium* infected male C3H/HeNCr mice	*C. rodentium* (bacteria) by oral gavage	KO in water emulsion	40 mg KO/Body weight (kg)	Gavage	26 days: 7-day treatment prior to and 19-day co-treatment with *C. rodentium*	↓ Colon index (Colon weight/body weight), Crypt length, *IL-1β*, Pparg, Gzmk, histamine ↑leap2, *Gas7*, *Muc6*, abundance of butyrate producing taxa (*Roseburia* and *Clostridium*), microbial interactions, oleic acid, ricinoleic acid, 4-(2-amino-3-hydroxyphenyl)-2,4-dioxobutanoic acid, γ-glutamylglutamic acid, 5-hydroxyindoleacetic acid, *Anaeroplasmataceae*, *Clostridiaceae*, *Christensenellaceae* (family level) Enriched pathways: cGMP-PKG signalling, calcium signalling, phospholipase D signalling, gap junction, and purine metabolism, pathways related to secretion	[66]

Abbreviations: CCL, chemokine ligand; CEL, carboxyethyllysine; cGMP-PKG, cGMP-dependent protein kinase or protein kinase G; CML, carboxymethyllysine; DAO, diamine oxidase; DHA, docosahexaenoic acid; DSS, dextran sulphate sodium; EPA, eicosapentaenoic acid; FAs, fatty acids; Gas 7, growth arrest specific 7; GSA, glutamate-1-semialdehyde; GSH, glutathione; Gzmk, granzyme K; KC/GRO, keratinocyte chemoattractant/growth-related oncogene; KO, krill oil; IL, interleukin; Leap2, liver-expressed antimicrobial peptide 2; LPS, lipopolysaccharides; MDA, malondialdehyde; NO, nitric oxide; OUT, operational taxonomic unit; PG, prostaglandin; PPAR-γ, peroxisome proliferator-activated receptor γ; Pparg1α, PPAR-γ coactivator 1α; Ptgs, prostaglandin synthase; SCFAs, short-chain fatty acids; Sirt 1, sirtuin 1; TLR, Toll-like receptor 4; SOD, superoxide dismutase; TNF, tumour necrosis factor; ZO-1, zonula occludens-1; ↑, increased, ↓, decreased.

## 8. Beneficial Effects of EPA and DHA in Intestinal Inflammation

**Figure 1:**
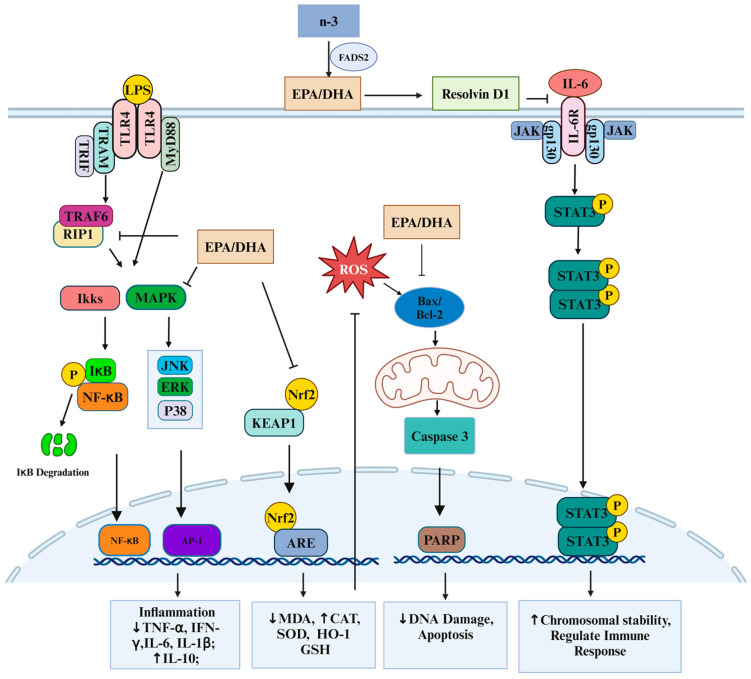
Schematic overview of the molecular pathways associated with the effects of EPA and DHA. Abbreviations: AP, activator protein; ARE, antioxidant responsive element; Bax, Bcl-2-associated protein x; Bcl-2, B-cell leukemia/lymphoma 2 protein; CAT, catalase; DHA, docosahexaenoic acid; ERK, extracellular signal-regulated kinases; EPA, eicosapentaenoic acid; FADS2, fatty acid desaturase 2; gp130, glycoprotein 130; GSH, glutathione; HO-1, heme oxygenase 1; IFN, interferon; IL, interleukin; IKK, IκB kinase; JAK, Janus kinase; JNK, c-Jun N-terminal kinase; KEAP1, Kelch-like ECH-associated protein 1; LPS, lipopolysaccharides; MAPK, mitogen-activated protein kinase; MDA, malondialdehyde; MyD88, myeloid differentiation primary response 88; NF-κB, nuclear factor kappa B; Nrf, nuclear respiratory factor; PARP, poly (ADP-ribose) polymerase; RIP, ribosome-inactivating protein; SOD, superoxide dismutase; STAT, signal transducer and activator of transcription; TLR, Toll-like receptor; TNF, tumour necrosis factor; TRAM, TIR domain-containing adaptor-inducing IFN-β-related adaptor molecule; TRAF, TNF-R-associated factor; TRIF, TIR-domain-containing adapter-inducing IFN-β; ↑, increased, ↓, decreased.

EPA and DHA are the two main LC n-3 PUFAs in KO. Several studies have shown that these fatty acids can improve the function and integrity of the intestinal barrier [136,137,138,139]. DHA administered at 30 mg/kg/day for seven days repaired gross morphological damage to the intestinal wall following inflammation and injury induced by DSS, including mucosal erosion, crypt structure changes (crypt branching and distortion), and goblet cell loss [136]. EPA and DHA have been shown to protect human colonic goblet cells by counteracting the reduction in Muc2 secretion triggered by palmitic acid, and thereby mitigating ER stress [140]. Additionally, EPA has been observed to elevate the levels of trefoil factor-3 (TFF-3), a factor primarily expressed in the goblet cells of both the large and small intestines, playing a vital role in the maintenance and restoration of intestinal mucosa, as well as the preservation of epithelial integrity [141]. Daily treatment with DHA at 35.5 mg/kg for two weeks can ameliorate changes to intestinal permeability in IL-10-deficient mice with spontaneous chronic colitis by increasing the expression of TJ-associated proteins, occludin, and ZO-1 [137]. Another study demonstrated that pre-treatment with DHA-enriched phospholipids (DHA-PL) or EPA-enriched phospholipids (EPA-PL) at 500 mg/kg/day for four weeks could decrease the Bax/Bcl-2 ratio and cleave caspase-3 protein levels in the ileum in an animal model of LPS-mediated intestinal barrier injury [139]. Bax, Bcl-2, and cleaved caspases-3 are pivotal regulators of apoptotic cell death. In addition, it was found that pre-treatment with DHA-PL or EPA-PL can increase the LC3-II/LC3-I ratio and Beclin1 protein expression, as well as decrease the levels of p62 protein to prevent LPS-induced defective autophagy in intestinal epithelial cells [139]. An in vitro study showed that EPA and DHA (6.25–25 μg/mL) treatments can improve the function and integrity of the intestinal barrier by promoting the localisation of TJ proteins to the plasma membrane and the expression of TJ proteins, ZO-1, Claudin-1, and Claudin, in deoxynivalenol (DON)-induced cell necrosis in intestinal porcine epithelial cells [138]. This study also demonstrated that both EPA and DHA decrease necroptosis-related protein expressions, including TNFR1, receptor-interacting protein (RIP)1, RIP3, dynamin-related protein 1 (Drp1), mixed lineage kinase domain-like (MLKL) phosphorylation, and phosphoglycerate mutase family member 5 (PGAM5) (Figure 1). A clinical study revealed that EPA (2 g/day for 90 days) improved endoscopic and histological inflammation in patients with UC [142]. The authors determined that EPA appears to play a protective role during UC remission by inhibiting signal transducer and activator of transcription 3 (STAT3) activation through suppressor of cytokine signaling 3 (SOCS3) transcriptional induction. Additionally, EPA modulated intestinal differentiation by inducing the expression of both HES1 and KLF4 proteins, ultimately leading to an increase in the number of goblet cells. Collectively, these findings suggest that the effect of EPA and DHA on intestinal cell injury and barrier function impairment may be related to the inhibition of the necroptosis and epithelial cell apoptosis signalling pathways, and the regulation of autophagy.

The antioxidant effects of DHA and EPA were demonstrated in mice with LPS-induced intestinal barrier injury; pre-treatment with 500 mg/kg DHA-PL and EPA-PL daily for four weeks effectively mitigated the LPS-induced increase in MDA level and decline in CAT and SOD activities, as well as the GSH content in the small intestine [139]. Furthermore, DHA-PL and EPA-PL pre-treatment increased nuclear levels of Nrf2 and HO-1 protein expression but reduced cytoplasmic Nrf2 levels, which were almost abolished. Notably, this reduction was nearly abolished by a sirtuin 1 (SIRT1)-specific inhibitor. Therefore, the authors reported that DHA-PL and EPA-PL alleviate intestinal oxidative stress via the SIRT1/Nrf2 pathway. Some studies have demonstrated that EPA and DHA can modulate the composition of the gut microbiota. Supplementation with 200 mg/kg DHA-PL or DHA-TG for 14 days in mice with DSS-induced colitis regulated gut microbial composition via a reduction in *Bacteroides* and an increase in *Odoribacter*, a SCFA-producing bacteria [143]. Additionally, a randomised trial revealed that 4 g/day of mixed DHA and EPA supplementation administered by either capsule and drink formulations for eight weeks induced a reversible increase in several SCFA-producing bacteria at the genus level, including *Bifidobacterium*, *Roseburia*, and *Lactobacillus* [144]. However, no significant alterations were observed in α or β diversity or the phylum composition associated with supplementation, which was considered to be a consequence of the short-term dietary intervention failing to overcome the dominant interindividual variation in the intestinal microbiome.

Various studies have demonstrated the anti-inflammatory effects of EPA and DHA to be associated with a decreased production of pro-inflammatory cytokines. In vitro studies have reported that DHA and EPA treatment can reduce pro-inflammatory cytokine production by regulating the NFκB signalling pathway in LPS-stimulated THP-1 monocyte-derived macrophages [145,146]. Interestingly, one study also showed that DHA treatment had no significant effect, when compared to the vehicle, on LPS–cell association, the cell surface expression of TLR4, the TLR4-myeloid differentiation factor 2 (MD2) complex, and CD14 in ultra-pure LPS-stimulated RAW 264.7 macrophages, while cytokines (IL-6 and TNF-α) were decreased. This observation implies that the anti-inflammatory effect of DHA may be exerted downstream of the activation of the TLR4 receptor [147].

In DSS-induced colitis, pre-treatment with DHA-PL and DHA-enriched triglyceride (DHA-TG) for 14 days downregulated pro-inflammatory IL-1β and TNF-α and upregulated anti-inflammatory IL-10 protein expressions [143]. Similarly, a seven-day DHA treatment reduced the gene expression levels of IL-1 β and TNF receptor superfamily member 1b (*Tnfrsf1b*) in mice with DSS-induced colitis [136]. Levels of TNFα, IFN- γ, and IL-17 were also shown to be suppressed in IL-10-deficient mice after 14 days of DHA treatment [137].

A study showed that a four-week pre-treatment with DHA-PL and EPA-PL downregulated the protein expressions of TNF-α, IL-1β, IL-6, inducible nitric oxide synthase (iNOS), and cyclooxygenase (COX)-2 in LPS-induced intestinal injury in mice [139]. Furthermore, DHA-PL and EPA-PL pre-treatment downregulated the LPS-mediated phosphorylation of protein IKKα/β, IκB-α, extracellular signal-regulated kinases 1/2 (ERK 1/2), c-Jun N-terminal kinase (JNK), and p38 induced by LPS [139]. These findings suggest that DHA-PL and EPA-PL ameliorate LPS-evoked intestinal inflammatory responses via the inactivation of the NF-κB and MAPK pathways. The detailed mechanisms involved in the positive effects of EPA and DHA, such as their anti-inflammatory and antioxidant effects, are shown in Figure 1.

It has been suggested that the lipid mediators derived from EPA or DHA metabolism may be potential targets for IBD treatment. The concentration of resolvin E1 (RvE1) is higher in patients with UC relative to CD patients, signifying its potential as a distinguishing marker for IBD classification during active disease. Conversely, during the remission phases of both diseases, the levels of protectin DX (PDX) were significantly higher in CD compared to UC [148]. Resolvin D1 (RvD1), a lipoxygenase (LOX) metabolite derived from DHA, has been shown to attenuate DSS-induced colitis by reducing the production of IL-6 [149]. Furthermore, RvD1 exerted an inhibitory effect on the IL-6-induced activation of JAK2/STAT3 signalling by blocking the assembly of the IL-6/IL-6R/gp130 complex, thereby suppressing IL-6-mediated chromosomal instability. Following EPA or DHA supplementation for two phases of 10 weeks in patients with chronic inflammation, it was observed that EPA reduces the gene expression of TNF-α in monocytes, while DHA decreases TNF-α, IL-6, MCP1, and IL-10. Further analyses indicated that EPA supplementation increases 18-HEPE and decreases the AA derivative, 15-HETE, while DHA supplementation increases 17-HDHA and 14-HDHA and reduces AA derivatives, including prostaglandin D2 (PGD2), prostaglandin E2 (PGE2), and thromboxane (TX) B2, compared to EPA. Interestingly, the reduction in TNF-α was achieved by EPA supplementation through the increase in multiple EPA derivatives, while DHA supplementation showed a stronger influence due to the reduction in AA derivatives. This observation highlights the potential mediating effects of plasma pro-resolving lipid mediators of LC n-3 PUFA on immunomodulatory actions. Correspondingly, the persistent activation and infiltration of neutrophils, which are characteristic of intestinal inflammation, were reduced in mice with DSS-induced colitis following treatment with lipid mediators (5,6-DiHETE) derived from DHA or EPA [136,137,143,150]. These findings suggest the role of derivatives of EPA and DHA during the active and remission phases of IBD.

In a clinical study, an inverse association between the development of CD and the dietary intake of DHA was reported [151]. Another study found that an intake of 500 mg/day of EPA for six months reduces clinical remission and calprotectin levels in UC patients [124]. Furthermore, an inverse association between the risk of UC and LC n-3 PUFA intake was reported [34]. In a recent mediation Mendelian randomisation study, it was reported that increased EPA has a causal effect in reducing the risk of IBD, but total n-3 FA, α-linolenic acid, and DHA exhibit limited protective effects against IBD risk [152]. The FADS2 gene is considered to be the likely central gene that regulates the effects of n-3 PUFAs on the risk of IBD, implying that desaturation steps in fatty acid metabolism may have a crucial role in the association between n-3 PUFAs and IBD. It also suggests the direct involvement of the liver in the pathogenesis of IBD [152]. Another clinical study found that IBD patients following an n-3 PUFA diet plan to achieve an n-3/n-6 ratio of ≈1 was beneficial in maintaining remission [153]. Conversely, two randomised, double-blind, placebo-controlled trials on patients with CD investigating the effects of 4 g/day n-3 FAs containing 50–60% EPA and 15–20% DHA for up to 58 weeks found no effective results for the prevention of relapse in CD compared with the placebo [154,155]. A recent review also reported that n-3 fatty acid supplementation does not improve inflammation in IBD patients [156]. Given that the evidence remains inconclusive, there is currently insufficient data to recommend a specific dose and duration for either EPA/DHA combination or EPA/DHA alone in IBD clinical research. Thus, further clinical investigations are required to elucidate the anti-inflammatory effects of DHA and EPA in IBD.

## 9. Beneficial Effects of Astaxanthin in Intestinal Inflammation

A small amount of astaxanthin is found in KO. Astaxanthin is an antioxidant that belongs to the xanthophyll class of carotenoids [157]. The colour of astaxanthin is a dark reddish orange, which likely contributes to the pinkish pigmentation of the native krill [158]. Due to its molecular structure comprising hydroxyl (OH) and keto (C=O) moieties on each ionone ring, astaxanthin has unique chemical properties, which include a higher antioxidant activity and a more polar nature than other carotenoids [158].

Diets enriched with astaxanthin have been reported to attenuate symptoms of IBD. In DSS-induced colitis, supplementation with 100 or 200 ppm astaxanthin for 17 weeks or 0.04% astaxanthin powder for ten days has been demonstrated to reduce the disease activity index and colon weight/length ratio, as well as restore the mucosal integrity disrupted during intestinal inflammation [159]. These clinical and morphological improvements were also observed in a more recent study following treatment with astaxanthin-enriched colon-targeted alginate particles (Ax-Alg) [160].

Possible mechanisms by which astaxanthin acts to attenuate IBD include the alleviation of colonic mucosal inflammation, reducing oxidative stress, and modulating gut microbiota dysbiosis. Astaxanthin significantly suppressed the mucosal mRNA expression of pro-inflammatory cytokines, including IL-1β, IL-6, TNF-α, IL-36α, IL-36γ, and COX-2, in DSS colitis [161]. In addition, astaxanthin suppressed the DSS-induced mucosal activation of MAPKs, including ERK1/2, p38, and JNK, and the translocation of NF-κB p65 and Activator protein (AP)-1 into the nucleus. These findings indicate that astaxanthin prevented the development of DSS-induced colitis via the direct suppression of the activation of the NFκB and MAPK pathways [161]. Ax-Alg was demonstrated to reduce the level of LPS and translocate it from the intestinal lumen to the blood in DSS-induced colitis, suggesting that astaxanthin may attenuate the inflammatory response in colitis by inhibiting LPS expression or translocation [160]. A recent study reported that astaxanthin succinate diester (Asta-SD) counteracted the downward trend of the F/B ratio and reduced the abundance of *Bacteroides*, *Geobacter*, *Alistipes*, *Butyrimionas*, and *Parabacteroides* in mice with DSS-induced colitis [162]. Treatment with Asta-SD also promoted the proliferation of beneficial bacteria *Anaerotruncus* and *Lactobacillus*. This observation is consistent with a previous study examining the effects of KO on DSS-induced colitis [64]. Asta-SD decreased the gene expression levels of TNF-α, IL-6, and IL-1β. Furthermore, this study also found that the abundance of *Bacteroides*, *Parabacteroides*, and *Butyrimionas* were positively correlated with the gene expression of IL-6 [162]. These findings suggest the Asta-SD ameliorates inflammation in an animal model of UC via the maintenance of intestinal microbiota homeostasis.

Astaxanthin treatment in DSS-induced colitis mice downregulated the cytoplasmic level of 8-hydroxy-2′-deoxyguanosine (8-OhdG), iNOS, and myeloperoxidase (MPO) in colon tissue while upregulating T-superoxide dismutase (T-SOD), which protects the mitochondria by removing uncharged H_2_O_2_ into the cytosol [91,161]. SODs protect tissue against oxidative damage under inflammatory and oxidative stress conditions in IBD. Activating iNOS can promote the intestinal epithelial cells, as well as neutrophils and macrophages, to produce superoxide and nitric oxide, which damage cytoskeleton proteins and result in a barrier disruption during mucosal inflammation [91]. Elevated MPO levels are associated with inflammation and increased oxidative stress, while 8-OHdG is a marker for endogenous oxidative damage to DNA [163,164]. Furthermore, it was also reported that astaxanthin attenuates inflammation in DSS-induced colitis by regulating intracellular enzymatic antioxidant activity to reduce ROS overload and mucosal injury [91].

## 10. Conclusions

In this review, the properties of KO and its bioactive components (EPA, DHA, and astaxanthin) and their therapeutic effects on IBD are discussed. Various in vitro and animal studies have demonstrated that KO can attenuate intestinal inflammation. Plausible mechanisms of the beneficial effects exhibited by KO include ameliorating the inflammatory response, reducing oxidative stress, improving the intestinal barrier, and modulating gut microbiota. However, the molecular mechanisms and pathways of its actions are not yet fully understood. Hence, future studies should focus on the molecular mechanisms underlying the health benefits of KO in IBD. The specific roles of KO’s bioactive components in protective, anti-inflammatory, and antioxidant activities in intestinal inflammation need to be further explored. Moreover, clinical studies on the efficacy of krill oil, either alone or in co-administration with the current therapy for IBD, would provide insight into the benefits of KO and its bioactive components for more effective IBD treatment.
